# Individualized Hemodynamic Management in Sepsis

**DOI:** 10.3390/jpm11020157

**Published:** 2021-02-23

**Authors:** Marcell Virág, Tamas Leiner, Mate Rottler, Klementina Ocskay, Zsolt Molnar

**Affiliations:** 1 Institute for Translational Medicine, Medical School, University of Pécs, 7624 Pécs, Hungary; viragmarcell@yahoo.com (M.V.); tamasleiner@gmail.com (T.L.); mate.rottler@gmail.com (M.R.); ocskay.klementina@gmail.com (K.O.); 2 Szent György University Teaching Hospital of Fejér County, 8000 Székesfehérvár, Hungary; 3 Anaesthetic Department, North West Anglia NHS Foundation Trust, Hinchingbrooke Hospital, Huntingdon PE29 6NT, UK; 4 Department of Anesthesiology and Intensive Therapy, Poznan University of Medical Sciences, 61-701 Poznan, Poland; 5 Department of Anesthesiology and Intensive Therapy, Markusovszky Teaching Hospital, 9700 Szombathely, Hungary; 6 Multidisciplinary Doctoral School, University of Szeged, 6720 Szeged, Hungary

**Keywords:** septic shock, hemodynamic monitoring, early goal-directed therapy, lactate, fluid therapy

## Abstract

Hemodynamic optimization remains the cornerstone of resuscitation in the treatment of sepsis and septic shock. Delay or inadequate management will inevitably lead to hypoperfusion, tissue hypoxia or edema, and fluid overload, leading eventually to multiple organ failure, seriously affecting outcomes. According to a large international survey (FENICE study), physicians frequently use inadequate indices to guide fluid management in intensive care units. Goal-directed and “restrictive” infusion strategies have been recommended by guidelines over “liberal” approaches for several years. Unfortunately, these “fixed regimen” treatment protocols neglect the patient’s individual needs, and what is shown to be beneficial for a given population may not be so for the individual patient. However, applying multimodal, contextualized, and personalized management could potentially overcome this problem. The aim of this review was to give an insight into the pathophysiological rationale and clinical application of this relatively new approach in the hemodynamic management of septic patients.

## 1. Introduction

Early, adequate hemodynamic stabilization remains the cornerstone of resuscitation of the critically ill. Fluid resuscitation and vasopressor support are the most frequent treatments used to avoid or treat hypoperfusion and maintain adequate oxygen delivery to the tissues. Theoretically, intravenous fluid administration can improve oxygen delivery (DO_2_) by increasing cardiac output (CO), and vasopressors can maintain adequate perfusion pressure. However, both treatments can have deleterious effects when used in an inappropriate manner: under-resuscitation may cause hypoperfusion and positive fluid balance could increase mortality, especially in acute respiratory distress syndrome (ARDS) or even in septic shock [1,2,3]. From a physiological standpoint, a coherence between macro- and microcirculation is necessary for adequate functioning [4]. Translating this into clinical practice means that whatever we do to correct systemic hemodynamics only makes sense if it is followed by improved microcirculatory perfusion and oxygen delivery. There have been several attempts to recommend the most feasible approach in hemodynamic management, but none of them has gained generalized acceptance as being superior to the others. One of the most investigated approaches is the so-called “goal-directed therapy” (GDT), which is a very broad term and by-and-large means that prespecified values of certain physiological indices are followed and compared to standard monitoring. Although goal-directed approaches have been tried and tested in several large trials, results are contradictory [5,6,7,8]. This could be due to the fact that these approaches mainly tried to optimize macrocirculatory indices according to fixed values and neglected the patients’ individual demand. To overcome this limitation, putting the available pieces of the “hemodynamic puzzle” (DO_2_, oxygen consumption (VO_2_), and tissue perfusion) together was proposed as an alternative approach [9]. Unfortunately, this concept is in contrast with the rather arbitrary and uncoordinated use of fluids by clinicians worldwide as demonstrated in the FENICE study [10]. However, the importance of individualizing resuscitation measures was also recommended by the recent European Society of Intensive Care Medicine task force [11]. 

The aim of this review was to give an insight into the pathophysiological rationale and clinical application of this relatively new approach in the hemodynamic management of the critically ill—including septic patients.

## 2. Paradigm Shift in Definitions

Although our title suggests sepsis-specific management, this may require some clarification. In 2016, sepsis was defined as “life threatening organ dysfunction caused by a dysregulated immune response to infection” [12]. The inclusion of the term “dysregulated immune response” was a fundamental and conceptual change compared to the long standing “systemic inflammatory response syndrome” [13]. Dysregulated host response means that the physiological balance between pro-, and anti-inflammation—which is fundamental for tackling and recovering from any type of injury (be it tissue trauma or infection)—is lost [14]. This imbalance is the result of an excessive release of pro-inflammatory mediators, causing a feature that is called a “cytokine storm”, “cytokine release syndrome”, or “hyperinflammation” [15]. The hemodynamic effects of a cytokine storm can lead to the loss of control of the vascular tone and may cause “vasoplegic shock”, which is identical to the condition that we see in septic shock. The only difference is that vasoplegic shock as a term can be applied in conditions when there is no infection, such as trauma, sterile inflammation, burns, etc., in which case the inflammation can be just as severe as—or even worse than—seen in sepsis. Discussing this in further detail is well beyond the scope of the current review, but it may become clear now that the message of the following paragraphs could be applied not only in sepsis, but in any critically ill conditions.

## 3. Hemodynamic Management Concepts

### 3.1. Goal-Directed Therapy 

According to the recent recommendations of the Surviving Sepsis Campaign Guidelines [16], after the first 3 h of a fixed fluid regimen of 30 mL/kg crystalloids, a goal-directed approach should be followed with frequent re-assessment, preferring dynamic variables over static ones to ensure adequate perfusion and a balance between oxygen delivery and demand. These statements were supported by low and moderate quality evidence [16]. The guideline strongly recommends a target mean arterial pressure (MAP) of 65 mmHg and advises to refrain from the use of central venous pressure (CVP) and other static goals.

Rivers et al. performed a pivotal randomized controlled trial in 2001 with 263 patients to evaluate the efficacy of early goal-directed fluid therapy (EGDT) [5]. Patients with septic shock or severe sepsis were enrolled. Standard care was guided by CVP, MAP and urine output. In the intervention group, central venous oxygen saturation (ScvO_2_) was continuously monitored for 6 h alongside the parameters used in standard therapy, aiming to maintain ScvO_2_ equal to or above 70%. As per the protocol, 500 milliliters of crystalloids was given every 30 min to maintain a CVP between 8 and 12 mmHg. In the case of lower-than-target ScvO_2_, red blood cells were transfused until hematocrit values reached 30%.

Significantly lower in-hospital mortality was found in the intervention group (30.5% versus 46.5%, *p* = 0.009). Physiologic parameters and Acute Physiology and Chronic Health Evaluation (APACHE) II scores in the first 24 h indicated that the goal-directed approach resulted in less severe organ dysfunction.

Later, three workgroups attempted to repeat these results with slight alterations in the protocol. However, neither the PROMISE, ARISE, nor PROCESS trials could prove goal-directed to be superior to standard resuscitation strategies [6,7,8].

PROMISE was a multicenter pragmatic randomized trial from England with 1260 enrolled patients and 90-day follow-up [6]. The relative risk of 90–day mortality was 1.01 in the EGDT group, and there were no significant differences in secondary outcomes, including quality of life and adverse events. Moreover, EGDT increased the cost of treatment. 

Similarly, the ARISE trial enrolled 1600 patients from 51 centers in Australia and New Zealand, with the same primary endpoint and non-significant results [7]. The PROCESS study was conducted at 31 centers in the United States, enrolling 1341 patients. Its primary endpoint was 60-day in-hospital mortality, which did not differ significantly in the two groups (21% for EGDT and 18.2% for standard therapy). Both 90-day and 1-year mortalities were similar [8].

The congruent conclusions of the three large multicenter studies highlighted that the results from the original study may have been biased. Furthermore, therapeutic advances from 2001 to 2014 could also have affected outcomes, as shown by the lower mortality rates of sepsis in general. The overall explanation at the time was that it could be due to the continuously updated Surviving Sepsis Guideline protocols [17]—including the new concept of a lower threshold of hemoglobin levels for transfusion, tighter blood glucose control, and the appearance of lung protective ventilation just to name a few; hence, the marginal advantage of early goal-directed therapy may have disappeared.

However, when we took a closer look at the mortality of septic shock reported in large observational studies, it has not really changed over the years and can be as high as 50% [18]; hence, there is still a lot of room for improvement. Therefore, one cannot exclude that the individualized approach of fluid management, which has strong pathophysiological rationale, could be more appropriate and may also improve outcomes in sepsis and septic shock.

### 3.2. Adding Measures of Oxygen Debt to the Picture 

The ultimate aim of hemodynamic management is to restore the balance between oxygen demand and supply at the tissue level. Outside clinical trials, it is not yet feasible to directly measure tissue perfusion; therefore, we desperately need surrogate markers of global oxygen extraction and tissue hypoxia that are readily available at the bedside to guide therapeutic interventions. Central venous oxygen saturation, venous-to-arterial carbon dioxide gap (dCO_2_), lactate, and capillary refill time have all been proposed as potential resuscitation targets in hemodynamically unstable patients.

ScvO_2_ is a frequently used blood gas parameter—as a surrogate of mixed venous saturation taken from a catheter inserted in the superior vena cava—and is routinely used in patients with any shock. ScvO_2_ is generally influenced by the hemoglobin level, oxygen saturation, dissolved oxygen, CO (components of DO_2_), and oxygen consumption. Changes in ScvO_2_ can potentially indicate clinically significant anemia, hypovolemia, and impaired myocardial function and can be affected by medications, body temperature, or a combination of any other factors that are able to influence the VO_2_/DO_2_ relationship [19]. It is known that both low and high ScvO_2_ values are associated with higher mortality in patients with sepsis [20,21]. Furthermore, it was recently demonstrated that persistently low ScvO_2_ was associated with a higher 90-day mortality in septic shock, independent from other risk factors for death [22]. Low ScvO_2_ is likely secondary to inadequate oxygen delivery and resuscitation [20], whereas supranormal ScvO_2_ values should be interpreted as insufficient oxygen uptake due to microcirculatory shunting or sepsis-induced mitochondrial dysfunction [23,24]. It is important to note that this feature could make the interpretation of ScvO_2_ extremely difficult at the bedside. Although the three previously mentioned large, prospective, and multicenter randomized studies (PROCESS, ARISE, PROMISE) [6,7,8] failed to demonstrate any mortality benefits of an ScvO_2_-based approach, this may have been due in part to the protocols which applied fixed values of certain parameters as targets for the whole study population and neglected the patients’ individual needs.

Another important marker of tissue metabolism is lactate, which is known as an easily measurable parameter of tissue hypoperfusion/hypoxia, often reflecting anaerobic metabolism. Moreover, it is a recommended resuscitation target in septic shock [16], and its peak concentration and persistent hyperlactatemia after resuscitation is regarded as an important prognostic factor of unfavorable outcomes in shock [25,26]. It is essential to highlight that according to the latest evidence, sepsis-related lactate production is not solely due to tissue hypoxia or hypoperfusion; therefore, lactate “clearance” (a term often used, although not clearance per se, but rather the degree of change in lactate levels) or high lactate in sepsis are not always a true reflection of impaired oxygen delivery and tissue hypoperfusion [21,27].

One single-center retrospective cohort study examined the relationship between lactate and ScvO_2_ and found that lactate had very little predictive ability for ScvO_2_ in the vast majority of critically ill patients; therefore, lactate should not be used interchangeably with ScvO_2_ as a marker of tissue hypoxia [28]. This is not surprising if we take into account that lactate is only considered pathological when it is high, while ScvO_2_ reflects abnormality both when low and when elevated. It is also crucial to bear in mind that fluid resuscitation on its own can have a diluting effect on lactate levels; therefore, it may give a false positive signal of improvement in this scenario. Hence, putting it in context with other hemodynamic indices is important.

Another simply obtainable blood flow-related variable is central venous-to-arterial carbon dioxide partial pressure difference (pCO_2_ gap or dCO_2_), which requires the parallel analysis of arterial and central venous blood gas samples. From a physiological standpoint, adapting the Fick principle to carbon dioxide production and elimination, the following equation describes the pCO_2_ gap [29]:

(1) $$P\left(v-a\right){\mathrm{CO}}_{2}=\frac{{\mathrm{VCO}}_{2}}{\mathrm{CO}}$$

where VCO_2_ is CO_2_ production, and CO is cardiac output. This clearly shows the indirect relationship between pCO_2_ gap and CO and explains why an increased pCO_2_ gap usually corresponds to low flow states. A very recent meta-analysis suggested that increased dCO_2_ (>6 mmHg) was associated with increased mortality, elevated lactate levels, and a lower cardiac index in critically ill patients [30].

In addition to laboratory parameters, clinical signs can also be extremely useful tools to assess hemodynamics. Therefore, assessing obvious vital signs, including changes in mental status, decreasing urine output, etc., are important signals to raise the alarm. Measurement of capillary refill time (CRT) is a widely used method to assess peripheral perfusion at the bedside and when it is prolonged (>3 sec), it usually indicates centralized circulation. Critically ill patients with abnormal CRT after fluid resuscitation were shown to have a significantly higher chance of worsening organ failure [31] and a persistently prolonged CRT after initial fluid resuscitation was associated with an adverse outcome in septic shock patients with elevated lactate levels [32]. The effect of CRT-guided resuscitation on 28-day mortality in septic shock compared to a lactate clearance targeted strategy was examined in the recent ANDROMEDA-SHOCK trial [33]. Unfortunately, this resuscitation strategy was not able to improve survival. Central venous oxygen saturation and dCO_2_ gradients were not significantly different between groups, but the CRT guided group showed improved sequential organ failure (SOFA) scores at 72 h and received significantly less resuscitation fluids within the first 8 h. However, the very same group compared CRT- to lactate-targeted resuscitation and found comparable effects on regional and microcirculatory flow parameters but a faster achievement of the predefined resuscitation target in the CRT group [34]. They also suggested that stopping fluids in patients with CRT ≤ 3 s is safe in terms of tissue perfusion.

Skin mottling also provides valuable information on skin perfusion, hence centralized circulation. A semi-quantitative evaluation of mottling on skin area extension on the legs can range from score 0 (no mottling) to score 5 (when it extends above the groin) and has been shown to be a good predictor of survival in septic shock [35,36].

In summary ScvO_2_, dCO_2_, lactate, and skin perfusion could be used as equally important surrogates and serve as helpful complimentary tools to guide our efforts in restoring normal tissue perfusion in the critically ill. It is also important to understand that no one parameter is appropriate as a single target to guide resuscitation. We need to put several pieces of the puzzle together in context to have a clearer picture in order to help us to individualize therapy.

### 3.3. The Individualized/Personalized Concept

**Figure 1:**
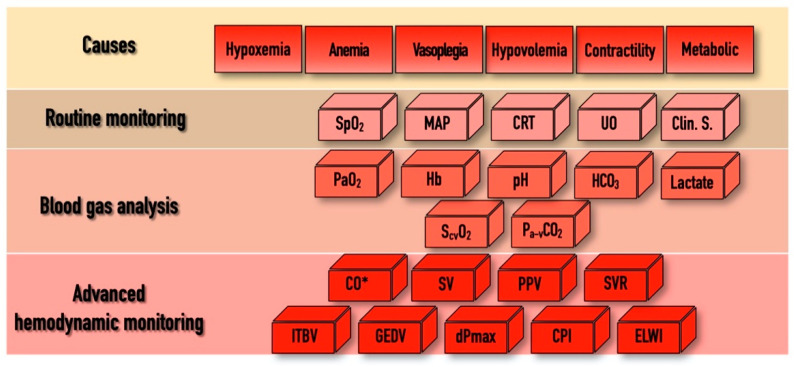
The “bricks” of individualized hemodynamic management. SpO_2_, pulse oximetry driven oxygen saturation; MAP, mean arterial pressure; CRT, capillary refill time; UO, urine output; Clin. S, clinical signs; PaO_2,_ partial pressure of oxygen; Hb, hemoglobin; HCO_3,_ bicarbonate; S_cv_O_2_, central venous oxygen saturation; P_a-v_CO_2_, arterial-to-venous carbon dioxide gap; CO, cardiac output; SV, stroke volume; PPV, pulse pressure variability; SVR, systemic vascular resistance; ITBV, intrathoracic blood volume; GEDV, global end-diastolic volume; dPmax, left ventricle contractility index; CPI, cardiac power index; ELWI, extravascular lung water index; * indicates that CO can be determined by invasive hemodynamic measurements or by echo-cardiography. For explanation, please see text.

In addition to the above-mentioned physiological indices, there are several other hemodynamic parameters, both static and dynamic, which were tested as resuscitation targets over the last few decades within the domain of “functional hemodynamic monitoring” [37]. This often required “advanced hemodynamic monitoring”, including the measurement of CO and other derived variables, as depicted in Figure 1. Despite the pathophysiological rationale of this approach, according to a recent meta-analysis, results of clinical trials were not convincing enough to enable any of the proposed algorithms to become standard practice [38]. The main limitations of these studies, as depicted in an editorial by Saugel et al., were heterogeneity in timing, the applied technology, choosing the right endpoint, and indeed the lack of personalization [39]. Furthermore, there were no prospective randomized trials that showed an effect for GDTon patient outcome and mortality when using advanced hemodynamic monitoring, whatever the technique used—be it echography [40], a pulmonary artery catheter [41], or transpulmonary thermodilution [42].

The only way to tailor hemodynamic support for the patients’ individual needs is to put the results of a detailed hemodynamic assessment, including components of macro-circulation, DO_2_ and VO_2_, into context. In a recent paper, this approach was named by Molnar et al. as a “multimodal, individualized, contextualized” concept [43]. The potential and most often used components of assessment, the “bricks” of this puzzle, are depicted in Figure 1. In practice, hemodynamic instability may occur for several reasons and in the combination of these, shown in the first row as “Causes”. As “Routine monitoring” records certain variables in a real-time fashion, it is highly likely that the first alarm signals will arrive from this domain. Blood gas analyses (both arterial and central venous) can help to confirm the severity of the alarm signal. In case of uncertainty, advanced monitoring can help to define the cause, severity, and the necessary interventions.

**Figure 2:**
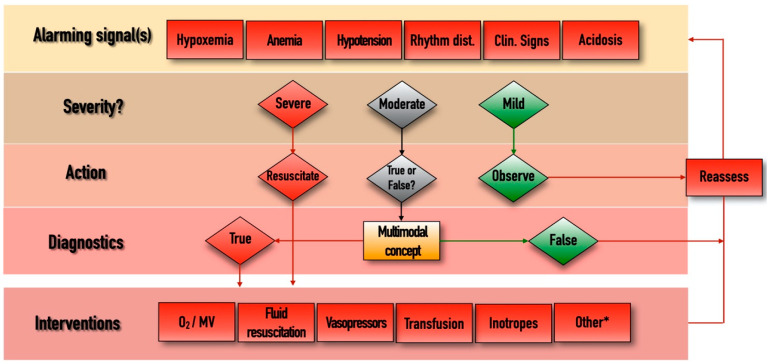
Management algorithm. Rhythm dist., cardiac rhythm disturbances; Clin, clinical; MV, mechanical ventilation. *, extracorporeal renal replacement therapies, immunomodulation, other adjuvant therapies. Whenever an “Alarming signal” is detected or suspected, the first step is to evaluate its “Severity”. If the signal is regarded as “Severe” (such as profound hypotension, extreme tachycardia, hypoxemia, etc.) then immediate resuscitation is needed in the form of the appropriate “Interventions”, after which the situation should be “Reassessed”, by checking the change in the alarming parameters and starting the loop again if necessary. If the alarm signal is regarded as “Mild”, then further observation and reassessment is enough. In cases of “Moderate” disturbances, when decisions cannot be made easily, the multimodal contextualized concept could become useful. This includes components listed in Figure 1, and putting these parameters in context can help us to determine whether the moderate alarm signal was indeed “True” or “False”. In cases of the presence of true pathology, the measures listed in the “Interventions” domain can be implemented, after which reassessment is again necessary.

The management algorithm is summarized and explained in Figure 2. The most obvious interventions to stabilize patients include oxygen therapy/mechanical ventilation, fluid resuscitation, vasopressor and/or inotropic support, and blood transfusion. Sometimes other (*) measures may also be needed, such as fluid removal by hemofiltration, renal replacement therapy, or immunomodulation including pharmacological therapy or extracorporeal blood purification. Putting all pieces of this puzzle into context may help choose the best and most appropriate therapy for hemodynamically unstable critically ill patients.

Undoubtedly, this concept is certainly complicated, requires well-trained personnel, and the monitors and disposables can be invasive and costly. These circumstances, and the uncertainty reported in clinical trials, are the main reasons this approach has not gained worldwide popularity and has not become routine management. However, the spread of recently developed less invasive and especially the non-invasive technologies that enable real-time, continuous evaluation of cardiac dynamics may change the current situation.

## 4. Future Perspectives

With the recent shift from static (such as MAP, CO, CVP, global end-diastolic volume—GEDV, etc.) to dynamic measurements (especially pulse pressure or stroke volume variation) [44], there might be a bright future for non-invasive hemodynamic monitoring. With the improvement of sensors and the development of new methods (e.g., the pulse contour algorithms), completely non-invasive techniques have emerged, combining the advantages of non-invasiveness with the precision of new-generation devices [45]. However, results on the accuracy of these technologies are controversial [46].

With the help of pulse contour algorithms, stroke volume and cardiac output can be calculated from an arterial blood pressure curve [45]. Several methods are available for these measurements, such as finger cuffs using the volume clamp method [47], radial artery applanation tonometry [48], and noninvasive brachial pulse wave analysis by hydraulic coupling [49].

Bioimpedance and bioreactance use similar principles, based on the fact that the cardiac chambers are electrically isolated [50]. They have been known for a long time, but their use in hemodynamic monitoring still has some limitations. Nevertheless, several devices are available using these two methods [50].

With the partial CO_2_-rebreathing technique, one can estimate CO with an applied and modified Fick principle using expired CO_2_ as an indicator. The Fick principle assumes that the blood flow (i.e., CO) through the alveoli equals the elimination of CO_2_ (VCO_2_) divided by the difference in venous-to-arterial CO_2_ [51]. It was first applied only in sedated and ventilated patients and required invasive blood sampling. More recently, a non-invasive model was developed, but it resulted in several limitations, such as face mask leaks introducing measurement bias, dependency on a steady respiratory state, and the necessity of adjusting for atelectasis and shunts [50].

Pulse wave transit time measurements only require an electrocadiogram and a plethysmograph to detect the time delay between the R wave and the peripheral pulse wave. Although these devices have several advantages, they must be calibrated frequently, and the algorithms are not yet capable of accounting for individual differences [52]. Pulse wave velocity is known for measuring arterial stiffness but is also being tested for continuous blood pressure measurements as well. One must take into account that this measure is age-dependent due to the general increase of arterial stiffness over time [53].

The above-mentioned technologies are certainly promising, but it is important to emphasize that the data we have at present are not robust enough to come to firm conclusions on the efficacy and patient benefit. However, new technologies are on the horizon, including regional perfusion and metabolic monitoring. Apart from potential new sensors and devices, visualization and handling of data are also rapidly developing fields. The availability of cross-checking measurements through device connectivity and more informed decision making by better visualization of data may transform hemodynamic monitoring in the near future [45].

The predictive capabilities of machine learning and artificial intelligence have also been tested in the field of hemodynamic monitoring with promising results. Hatib et al. reported that their algorithm was able to predict hypotension from arterial waveforms 15 min earlier with 88% specificity and 87% sensitivity. Five minutes before the onset of hypotension, these values were 92% and 92% with a 0.97 area under the curve [54]. The algorithm proved to be superior to other hemodynamic measurements in patients undergoing major surgery [55]. Artificial intelligence has performed remarkably in decision making when tested on patients with sepsis. Those patients who received the amount of intravenous fluids and dose of vasopressors calculated with the artificial intelligence tool had the lowest mortality in the tested septic cohort [56]. However, there is a long road ahead until big data and artificial intelligence become a bedside tool to help decision making, but it is certainly something with great potential to improve care in the future.

## 5. Conclusions

Hemodynamic support remains the cornerstone in the management of the critically ill regardless of its cause. However, all resuscitation measures (fluids, catecholamines, blood, etc.) can have harmful effects if they are under- or over-used. Therefore, being the most effective and causing the least harm concurrently should be of utmost importance. Personalizing therapy is a very intriguing and promising approach and can help us to deliver “as much treatment as needed”. At the moment, it seems complicated, sometimes invasive and costly, but hopefully in the long run, new technologies will help us to make it our usual routine. Until then, this multimodal, contextualized approach provides an excellent training tool for junior doctors by helping them to unveil pathophysiology at the bedside and also to understand the rationale of their actions. By doing so, they will hopefully understand the utmost importance of personalized medicine while treating the critically ill.
